# How digital mindfulness training promotes meaning in life among social workers: a parallel mediation analysis and latent profile analysis

**DOI:** 10.3389/fpsyt.2025.1700358

**Published:** 2025-11-21

**Authors:** Chang Rui, Jun Cao, Fuqiang Tan

**Affiliations:** Institute of Tourism Research, Huainan Normal University, Huainan, Anhui, China

**Keywords:** digital mindfulness training, meaning in life, social workers, perceived social exclusion, latent profile analysis

## Abstract

**Introduction:**

This study investigates the mechanisms by which digital mindfulness training promotes meaning in life among social workers and explores individual heterogeneity in this process.

**Methods:**

A parallel mediation model was used to test the pathways from digital mindfulness training to meaning in life. Latent Profile Analysis (LPA) was employed to identify distinct subgroups among the participants.

**Results:**

All five proposed hypotheses received strong empirical support. Our analysis revealed that social workers who participated in digital mindfulness programs reported a stronger meaning in life. The parallel mediation model identified two distinct pathways: digital mindfulness training was associated with (1) reduced perceived social exclusion and, separately, (2) fewer psychotic-like experiences. Both of these factors, in turn, independently contributed to a greater meaning in life. The LPA identified five distinct subgroups: the "Flourishing Professional," "Socially Isolated yet Resilient," "Typical Practitioner," "Internally Struggling but Connected," and "High-Risk and Distressed." These groups differed significantly in meaning in life, with the "High-Risk and Distressed" category scoring the lowest.

**Discussion:**

Viewed together, these patterns form a coherent explanatory structure that helps clarify how mindfulness interventions may operate. The findings also point to the importance of tailoring program content and delivery to address the particular vulnerabilities and strengths of each subgroup, rather than relying on a single, generic format.

## 1 Introduction

Social workers operate at a demanding crossroads where the turbulence of broad social change meets the urgent needs of individuals (1). Their work—anchored in the steady delivery of empathy and sustained emotional presence—can be transformative for clients seeking stability and direction. Yet the very qualities that enable this impact may, over time, erode practitioners’ own sense of purpose, leaving them more susceptible to burnout and, in some cases, prompting an exit from the profession (2). The recent growth of digital platforms has created opportunities to provide psychological support on a larger scale, including mindfulness−based programs tailored to sustaining the well−being of helping professionals. Whether these formats can truly deepen a meaning in life remains uncertain (3). Their influence may hinge not only on program content but also on how individual traits and cognitive−emotional patterns interact with the complex realities of social work. This includes dispositions toward psychotic-like symptoms—subclinical experiences such as heightened paranoia or unusual perceptual disturbances that, while not meeting the criteria for a formal psychotic disorder, are often exacerbated by chronic stress. Rather than assuming a single formula will serve all, the present study investigates the specific routes through which digital mindfulness training might bolster—or, in some contexts, fail to bolster—meaning in life for different subgroups of social workers (4).

Recent studies have increasingly pointed to the promise of digital mindfulness programs in reducing stress and anxiety among a wide span of users—from university students to frontline healthcare workers (5). While these findings are encouraging, they leave an important question unresolved: can such interventions influence the deeper construct of meaning in life? In the landscape of digital well-being research, social workers remain something of an afterthought, despite the fact that their everyday work exposes them to intense existential strain through repeated encounters with trauma, systemic inequities, and human distress (6). When they are included in studies, the analysis too often treats them as a single, undifferentiated group, overlooking the possibility that their baseline psychological resources, levels of burnout, and existential concerns may differ sharply. By collapsing these distinctions, earlier research risks obscuring meaningful subgroup patterns—patterns that could be crucial in shaping how individuals respond to digital mindfulness training (7). Addressing this gap calls for a more refined, person-centered perspective capable of capturing the diversity within the profession.

To bridge these notable research gaps, the current study offers a comprehensive examination of how digital mindfulness interventions influence social workers’ perceptions of meaning in life in nuanced ways (8). Leveraging cross-sectional survey data collected from a sizeable cohort of social workers in China—where the profession is undergoing rapid transformation but issues of practitioner well-being often receive insufficient attention—we adopt a dual-method analytical approach. The first stage applies Latent Profile Analysis (LPA) to transcend conventional mean-based comparisons, enabling the identification of distinct subpopulations characterized by different patterns of response to digital mindfulness training (9). In doing so, we directly address the question of which practitioners derive the greatest benefit from such interventions. Second, building on this person-centered foundation, we test a parallel mediation model to unravel the underlying mechanisms. This model examines the dual pathways through which the intervention fosters meaning in life: concurrently building psychological resources through the cultivation of self-compassion while also preserving them by mitigating professional burnout (10). By integrating these advanced methodologies, this study aims to provide a granular, empirically-grounded understanding that can inform the development of more personalized and effective well-being support for this vital professional community.

This study is poised to make several significant contributions to both theory and practice. Theoretically, our research extends the Conservation of Resources (COR) theory by delineating the nuanced pathways of resource management (11). First, by distinguishing between latent profiles, we show that a social worker’s initial “resource caravan” plays a decisive role in shaping their ability to capitalize on mindfulness training, clarifying who is most likely to initiate a positive resource gain spiral. Second, our parallel mediation analysis uncovers a dual mechanism underlying this process (12). Mindful attention and awareness function as a versatile resource management strategy operating on two fronts: they foster a resource−building pathway by sharpening the perception of pleasant life experiences (PLE), while simultaneously supporting a resource−preservation pathway by alleviating the harmful influence of perceived social exclusion (SE) (13). This dual−pathway framework offers a nuanced extension of Conservation of Resources (COR) theory (14). It illustrates that a single intervention may operate on two fronts: fostering the development of novel psychological resources, while simultaneously mitigating the erosion of social and relational assets. In this way, it contributes to the preservation—and possible enrichment—of a broader, higher−order resource, namely meaning in life. From an applied perspective, the findings suggest a shift away from generalized well−being programs toward more tailored strategies. Within such a framework, mindfulness training can serve distinct functions: acting as a catalyst to enhance positive affect among practitioners with limited capacity for pleasure, or functioning as a safeguard against the deleterious effects of social isolation (15).

## 2 Theoretical background and hypotheses

### 2.1 Conservation of resources theory

Social work takes place in an environment where resources—both personal and external—must be constantly balanced against persistent emotional demands and intricate social responsibilities (16). In such settings, a practitioner’s resilience is repeatedly tested, sometimes to its limits. To analyze these dynamics, the present study employs Conservation of Resources (COR) theory, not merely as contextual background, but as the central framework for generating hypotheses that can be empirically examined. COR theory advances two ideas that are highly pertinent here. The first is that losing personal or social resources typically causes a stronger psychological impact than gaining an equal amount, making the experience of depletion particularly harmful (17). The second is that building new resources often depends on using those already available—an investment that, if effectively managed, can trigger a self−reinforcing “gain spiral” over time.

From this perspective, meaning in life is conceptualized not just as an outcome, but as a cornerstone resource—a high-order, stable asset that provides resilience against future loss (18). Our study is therefore guided by the central question: How can social workers strategically manage their resources to protect and enhance this cornerstone resource? We posit that mindful attention (MA) acts as a meta-cognitive resource-management strategy. Its value lies in its dual capacity to both preserve existing resources and generate new ones, aligning perfectly with a parallel mediation model (19).

Specifically, we theorize a resource preservation pathway: Mindful attention helps to sever the automatic link between external events and internal distress, thereby mitigating the perception of social exclusion (SE)—a potent threat that drains crucial social-relational resources (20). This aligns with the “primacy of resource loss” principle, suggesting that preventing this loss is a primary mechanism for well-being. Concurrently, we theorize a resource generation pathway: By investing attentional resources into the present moment, individuals enhance their capacity to notice and savor pleasant life experiences, effectively generating new experiential resources (21, 22). This aligns with the “resource investment” principle, initiating a gain spiral toward meaning. Moreover, COR theory’s concept of “resource caravans”—the notion that resources cluster and the resource-rich get richer—provides a direct theoretical mandate for our use of Latent Profile Analysis (LPA). We expect to find distinct subgroups (i.e., resource caravans) of social workers, and we hypothesize that their pre-existing resource levels will determine their differential capacity to engage these preservation and generation pathways (23).

### 2.2 Digitalized mindfulness and meaning in life

At its core, mindfulness is a state of non-judgmental, present-moment awareness cultivated through specific attentional exercises. For social workers, who constantly operate in high-stakes environments, the cognitive ‘autopilot’ that often accompanies high-stress professions is not a luxury but a liability, leading to emotional exhaustion and a disconnection from their professional calling (24). Digitalized mindfulness training emerges here as a highly accessible, low-stigma intervention. From a COR theory perspective, it functions as a meta-cognitive skill that serves as a powerful resource-management tool. The practice of mindfulness training prompts a crucial ‘resource reallocation’ (25). It trains individuals to disengage from the resource-draining cycles of rumination and secondary traumatic stress, thus preserving valuable cognitive and emotional resources (3). More importantly, this conserved energy can then be reinvested into discerning the deeper significance of their work. By stepping off the treadmill of reactive crisis management, practitioners are afforded the mental space to reconnect with the profound sense of purpose and human connection that form the cornerstone of their professional calling (26). This process—shifting from a state of resource depletion to one of resource reinvestment in value-aligned activities—is the very mechanism through which a higher meaning in life in life can be forged. Therefore, we propose our first hypothesis:

H1: *Engagement with digitalized mindfulness training is positively associated with social workers’ meaning in life.*

### 2.3 The mediating role of social exclusion

For professionals dedicated to fostering social inclusion, the experience of social exclusion—whether from clients, colleagues, or the wider system—is not merely a professional setback but an existential threat (27). Social exclusion refers to the pervasive perception of being ostracized, devalued, or separated from one’s professional or social community. Within COR theory, this perception acts as a corrosive drain on vital social-relational resources, such as a sense of belonging and professional esteem, triggering a potent resource loss cycle. Herein lies the protective power of mindfulness (3). Digitalized mindfulness training serves as a cognitive buffer, fostering a state of decentering. This allows practitioners to observe rejecting social cues or critical feedback non-reactively, as transient mental events rather than as definitive indictments of their self-worth (28). By creating this crucial psychological space, mindfulness disrupts the automatic pathway from a negative social event to the debilitating feeling of exclusion, effectively staunching the loss of relational resources at its source (29). A meaning in life, in turn, is deeply contingent on the fundamental human need for relatedness and belonging. When this foundation is protected from the erosion of social exclusion, meaning can be sustained and even flourish. Thus, we posit that mindfulness does not create meaning out of a vacuum; rather, it protects the relational soil in which meaning can grow. This constitutes a critical resource preservation pathway (30). Accordingly, we hypothesize:

H2: *Perceived social exclusion mediates the positive relationship between engagement with digitalized mindfulness training and social workers’ meaning in life.*

### 2.4 The mediating role of psychotic-like experiences

The relentless exposure to trauma, systemic dysfunction, and human suffering places social workers at a unique risk for the fraying of their own cognitive fabric. This can manifest as Psychotic-Like Experiences (PLEs): subclinical, transient episodes of perceptual distortions, intrusive thoughts, or mild paranoia that erode an individual’s stable sense of reality (31). From a COR theory perspective, PLEs represent a profound resource drain, consuming vast cognitive and emotional energy as individuals struggle to distinguish internal noise from external reality. This internal chaos makes the construction of a coherent life narrative—a prerequisite for a meaning in life—nearly impossible. Meaning cannot be built upon the shifting sands of a destabilized mind (32).

Here, digitalized mindfulness training intervenes not as a mere coping skill, but as a fundamental tool for cognitive stabilization (33). Through the practice of cognitive defusion, mindfulness teaches practitioners to observe these intrusive thoughts and perceptions with detached awareness, recognizing them *as* mental events rather than *as* reality itself (34). This act of non-judgmental observation functions as a circuit breaker, halting the escalation of PLEs and preserving the critical resource of cognitive integrity (35). By reducing this “cognitive static,” mindfulness restores the mental clarity and internal coherence necessary for an individual to engage in the higher-order process of meaning-making. In essence, mindfulness does not directly bestow meaning; instead, it quiets the internal storm, providing the stable ground upon which a meaningful life can be built (36). This represents a foundational resource preservation pathway. Therefore, we hypothesize:

H3: *Psychotic-like experiences mediate the positive relationship between engagement with digitalized mindfulness training and social workers’ meaning in life.*

### 2.5 A person-centered approach

The pathways described above, while theoretically sound, implicitly assume a homogenous population of social workers who respond to interventions in a uniform manner. However, this variable-centered approach may mask a more complex reality. We challenge this assumption, positing that social workers are not a monolith (37); they enter the professional field with vastly different “resource caravans,” as conceptualized by COR theory. To capture this critical heterogeneity, we adopt a person-centered approach using Latent Profile Analysis (LPA). We expect to identify distinct subgroups of social workers characterized by unique constellations of their initial resources and vulnerabilities (e.g., levels of burnout, self-compassion, and social support) (38).

Specifically, we anticipate the emergence of at least three profiles: a “Resilient” profile, characterized by high resources and low vulnerabilities; a “Vulnerable” profile, marked by depleted resources and high vulnerabilities; and an “At-Risk” or “Struggling” profile, representing an intermediate state (39). These profiles are not merely descriptive categories; they represent fundamentally different starting points for resource management and meaning-making. An individual’s capacity to engage in the resource generation and preservation pathways we previously outlined is likely contingent upon which profile they belong to (40). Consequently, we expect these naturally occurring subgroups to exhibit significant differences in their baseline meaning in life. This leads to our final set of hypotheses:

H4: *There are distinct and meaningful latent profiles of social workers based on their initial levels of psychological resources and professional vulnerabilities.*
H5: *These latent profiles will significantly differ in their reported meaning in life, with the ‘Flourishing Professional’ profile exhibiting the highest level and the ‘High-Risk and Distressed’ profile exhibiting the lowest level.*

## 3 Methods

### 3.1 Participants and procedure

Participants were social workers recruited via convenience sampling from professional online platforms in China. A recruitment advertisement was posted on various social work-related online communities, forums, and social media groups (e.g., WeChat groups), which explained the study’s purpose, estimated completion time, and voluntary nature, and provided a link to the online survey. Upon accessing the survey, participants in the intervention group were first guided through the digital mindfulness training before proceeding to the questionnaire. This intervention consisted of a structured, 15-minute, pre-recorded audio-guided exercise, developed by the research team based on the core principles of Mindfulness-Based Stress Reduction (MBSR; 41) and narrated by a calm female voice. The session began with a brief period for settling in (minutes 1-3), followed by a core exercise focused on breath awareness, during which participants were guided to non-judgmentally observe their breath and gently return their attention when the mind wandered (minutes 3-10). The exercise then transitioned to a brief body scan (minutes 10-13) before concluding with guidance on returning awareness to the external environment (minutes 13-15). Prior to starting the audio, participants were instructed to find a quiet space and use headphones for an optimal experience. This standardized protocol was designed to ensure intervention fidelity and provide a replicable mindfulness experience, after which participants immediately proceeded to complete the survey measures. The inclusion criteria were as follows: (a) being at least 18 years of age; (b) currently employed as a full-time social worker in mainland China with a valid professional qualification certificate; and (c) having at least one year of full-time professional experience in social work. The procedure was structured as follows. Upon clicking the survey link, participants were first presented with an electronic informed consent form. This form detailed the research objectives, data usage, confidentiality protocols, and their right to withdraw at any point without penalty. After providing consent, they proceeded to the initial section of the questionnaire, which served as a screening tool to verify that they met all three inclusion criteria. Eligible participants then continued to complete the full battery of scales. To ensure data quality, attention check items (e.g., “For this item, please select ‘Strongly Agree’”) were embedded within the survey. An initial sample of 900 participants was recruited. Following data collection, the raw data underwent a rigorous screening process. A total of 49 responses were excluded due to incomplete submissions from midway withdrawal, failure to pass the attention check items, or not meeting the inclusion criteria. Consequently, the final valid sample consisted of 851 participants, yielding an effective response rate of 94.6%.

### 3.2 Measures

To measure trait mindfulness, we administered the Mindful Attention Awareness Scale (42). This 15-item instrument requires respondents to rate the frequency of experiences related to inattention and automatic behavior on a 6-point scale (1 = Almost Always to 6 = Almost Never). The average score across all items was used for analysis, with higher scores indicating greater mindfulness.

Psychotic-like experiences (PLEs).To assess psychotic-like experiences (PLEs), we utilized the 8-item positive symptom subscale from the Community Assessment of Psychic Experiences (CAPE-P8; (43)). This instrument gauges the frequency of six delusional and two hallucinatory symptoms within the past month. Participants provided their responses on a 4-point frequency scale (1 = Never to 4 = Almost Always). We then calculated a total score by summing the items (range: 8–32), where higher scores reflect a greater frequency of PLEs.

Social exclusion. Perceived social exclusion was measured with the 11−item scale developed by (44). The instrument taps into feelings of being marginalized or disregarded in social interactions. Items include statements such as “I feel ignored by the people around me,” which participants rated on a 7−point Likert−type scale ranging from 1 (Strongly Disagree) to 7 (Strongly Agree). Item responses were averaged to generate an overall score, with higher values reflecting a greater sense of social exclusion.

Meaning in life. Meaning in life was assessed using the Presence of Meaning subscale of the Meaning in Life Questionnaire (MLQ; (45)), consisting of five items. This subscale captures the extent to which participants perceive their lives as meaningful. Sample items include “I understand my life’s meaning.” Ratings were collected on a 7−point scale from 1 (Absolutely Untrue) to 7 (Absolutely True), and the mean of these ratings was used as the index, with larger scores indicating a stronger perceived presence of meaning.

## 4 Results

### 4.1 Latent profile analysis results

Latent Profile Analysis (LPA) was carried out with the tidyLPA package in R (version 4.3.2). The analysis aimed to classify participants into empirically derived subgroups based on two key indicators: psychotic−like experiences (PLEs) and perceived social exclusion (SE). This approach was selected to detect naturally occurring patterns in the joint distribution of these variables, rather than relying on *a priori* categorical assumptions. To determine the optimal number of profiles, we compared models with one to six classes based on multiple criteria: lower values for the Akaike Information Criterion (AIC), Bayesian Information Criterion (BIC), and sample-size adjusted BIC (SABIC); higher Entropy values (>.80); and a significant Bootstrapped Likelihood Ratio Test (BLRT; *p*<.05). The final decision was also informed by model parsimony and the theoretical interpretability of the profiles.

### 4.2 Model selection

The model fit indices for each solution are presented in **Table 1**. As shown, the AIC, BIC, and SABIC values generally decreased as the number of profiles increased, indicating a better fit with more complex models. Specifically, the BLRT results revealed that, with the exception of the three-profile solution, each model from two to six profiles offered a significant improvement in fit over the preceding (*k*−1) model (all *p*s <.01). However, the transition from a two- to a three-profile solution was not statistically significant (BLRT *p*= .129). Furthermore, the three-profile solution yielded a relatively low Entropy value of 0.692, suggesting it was not a robust classification.

**Table 1:** Fit indices for latent profile models with different numbers of profiles.

Classes	LogLik	AIC	BIC	SABIC	Entropy	BLRT val	BLRT p
1	-22933.31	45942.62	46122.99	46002.31	1.000	NA	NA
2	-22508.57	45133.14	45408.43	45224.24	0.796	849.488	0.010
3	-22489.20	45134.39	45504.61	45256.91	0.692	38.742	0.129
4	-22275.95	44747.91	45213.06	44901.84	0.790	426.487	0.010
5	-22184.41	44604.82	45164.89	44790.16	0.797	183.092	0.010
6	-22072.09	44420.18	45075.19	44636.94	0.820	224.632	0.010

LogLik, Log-Likelihood; AIC, Akaike Information Criterion; BIC, Bayesian Information Criterion; SABIC, sample-size adjusted Bayesian Information Criterion; BLRT, Bootstrapped Likelihood Ratio Test.

Among the remaining candidate models, while the four-profile model had an adequate Entropy of.790, the five-profile (.797) and six-profile (.820) solutions demonstrated superior classification clarity. When comparing the five- and six-profile solutions, we observed that although the six-profile model had the lowest BIC and the highest Entropy, it was rejected on the grounds of parsimony. Specifically, it produced smaller subgroups (9.6% and 9.3% of the sample, respectively), which could compromise the stability and theoretical interpretability of the profiles. In contrast, the five-profile solution demonstrated excellent overall fit (AIC=44604.82, BIC=45164.89, SABIC=44790.16), good classification quality (Entropy = .797), and represented a significant improvement over the four-profile model (BLRT *p*<.01). Therefore, balancing model fit, parsimony, and theoretical interpretability, we selected the five-profile solution as the optimal model for subsequent analyses.

**Figure 1:**
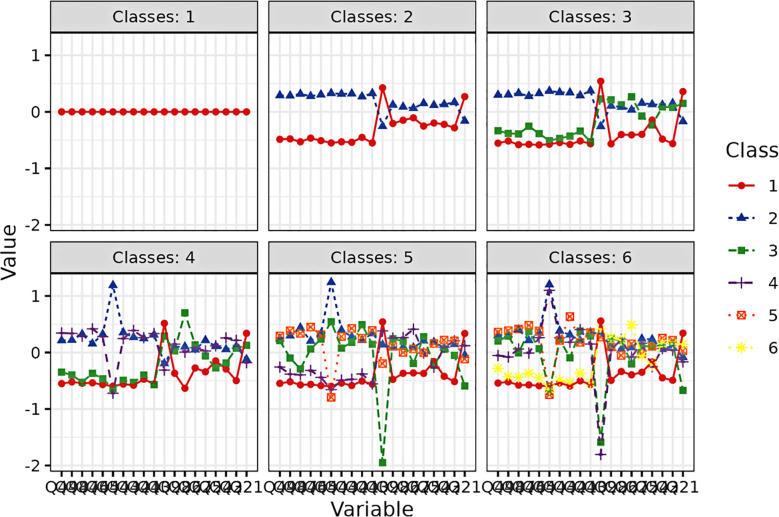
Latent class profile plot.

**Figure 2:**
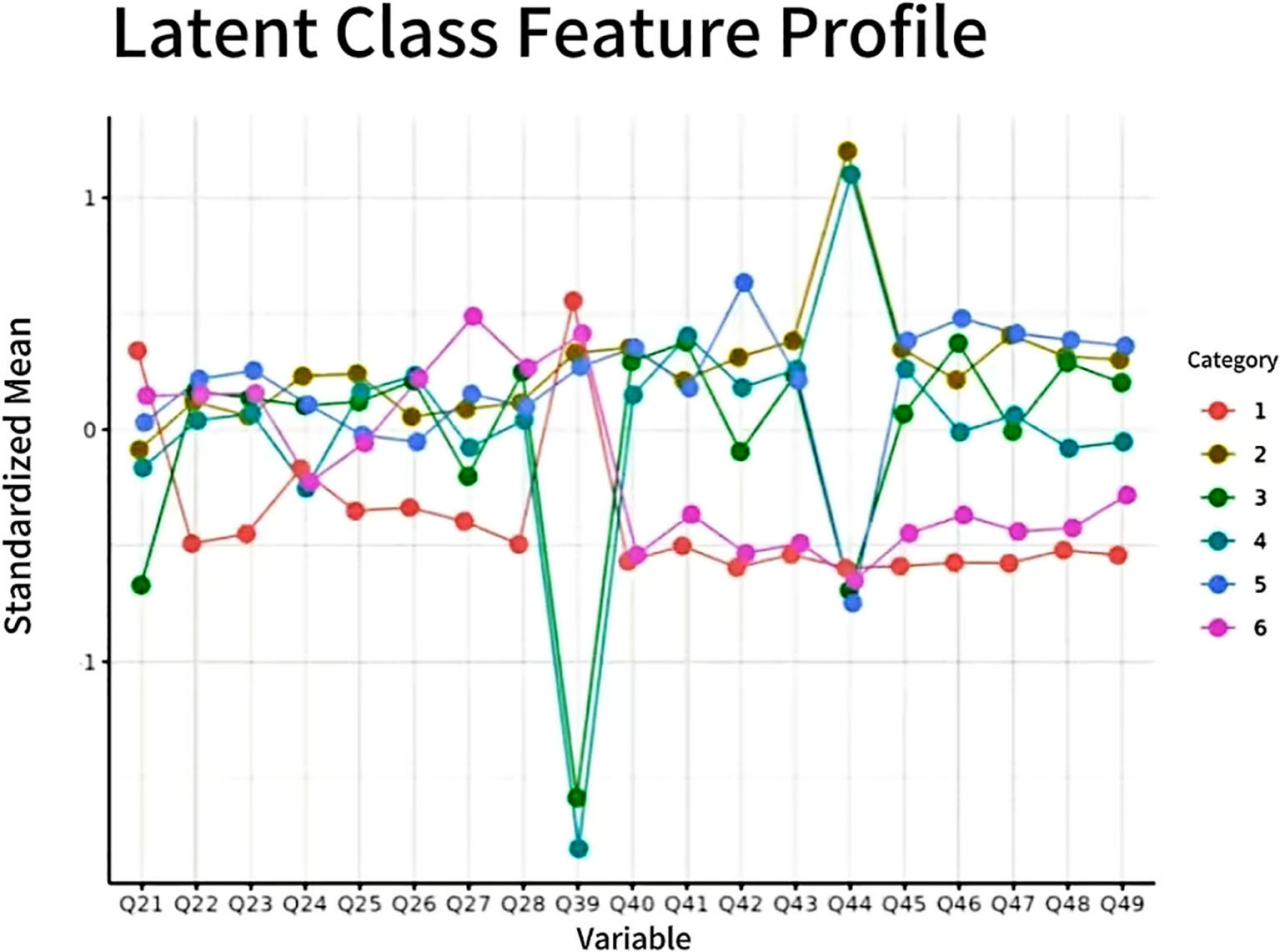
Latent Class Feature Profile.

Below are the results of the model-fitting curves **Figures 1**, **2**.

### 4.3 Characteristics of the latent profiles

The selected five-profile model revealed five distinct subgroups of social workers, each characterized by a unique combination of perceived social exclusion (SE) and psychotic-like experiences (PLEs). A fundamental premise of our interpretation is that lower scores on both the SE and PLEs scales indicate better psychosocial functioning. Based on their scoring patterns, these profiles were re-evaluated, named, and are described below in order from most adaptive to most at-risk.

C1: The Flourishing Professional (formerly C4, 14.2%): This profile represents the most adaptive and psychologically healthy subgroup. Characterized by the lowest scores on both social exclusion and psychotic-like experiences, these social workers demonstrate robust psychosocial functioning. They feel well-integrated within their professional environments (low SE) and maintain high levels of mental clarity and well-being (low PLEs). This pattern suggests a group of practitioners who have successfully cultivated supportive networks and effective coping mechanisms, enabling them to thrive in their demanding roles.
C2: The Socially Isolated yet Resilient (formerly C3, 8.8%): This subgroup presents a unique and important pattern: markedly high scores on social exclusion paired with very low scores on psychotic-like experiences. These practitioners appear to be mentally resilient and maintain psychological stability (low PLEs) despite experiencing significant professional marginalization, conflict, or a lack of belonging (high SE). They may be effective practitioners who are unfortunately situated in unsupportive or hostile work environments. Their resilience in the face of social adversity is a key characteristic.
C3: The Typical Practitioner (formerly C2, 30.0%): Constituting the largest subgroup, these social workers scored near the sample average on both dimensions. Their profile reflects the normative experience of the profession: a manageable level of psychological distress (moderate PLEs) co-occurring with a moderate degree of social and systemic friction (moderate SE). They are navigating the standard challenges and rewards inherent in social work without falling into extreme distress or flourishing unimpeded.
C4: The Internally Struggling but Connected (formerly C1, 22.9%): This profile reveals a paradoxical pattern of low social exclusion but high psychotic-like experiences. On the surface, these social workers appear well-connected and integrated into their teams (low SE). However, they internally experience a high frequency of distressing psychological symptoms, such as intrusive thoughts or perceptual anomalies (high PLEs). This suggests a group that may be adept at maintaining a professional facade while privately struggling with significant mental health challenges. Their difficulties are primarily internal rather than social.
C5: The High-Risk and Distressed (formerly C5, 24.0%): This profile represents the most vulnerable subgroup, characterized by high scores on both social exclusion and psychotic-like experiences. These social workers are facing a dual crisis: they feel actively isolated and rejected by their professional environment (high SE) while simultaneously battling severe psychological distress (high PLEs). This combination points to a state of acute burnout, alienation, and significant risk for adverse mental health outcomes. This group requires the most urgent and intensive support.

In summary, this Latent Profile Analysis successfully identified five clinically meaningful and distinct patterns of psychosocial functioning among social workers. These findings move beyond a one-size-fits-all understanding of professional well-being, revealing a significant heterogeneity ranging from the flourishing to the critically at-risk. This empirical typology provides a crucial foundation for developing targeted support strategies (25). For instance, the “High-Risk and Distressed” group requires immediate, comprehensive intervention addressing both their social environment and psychological symptoms. In contrast, interventions for the “Socially Isolated yet Resilient” group should focus primarily on improving workplace integration and resolving interpersonal conflicts, as their internal coping resources appear to be intact.

### 4.4 Results of the parallel mediation model

**Figure 3:**
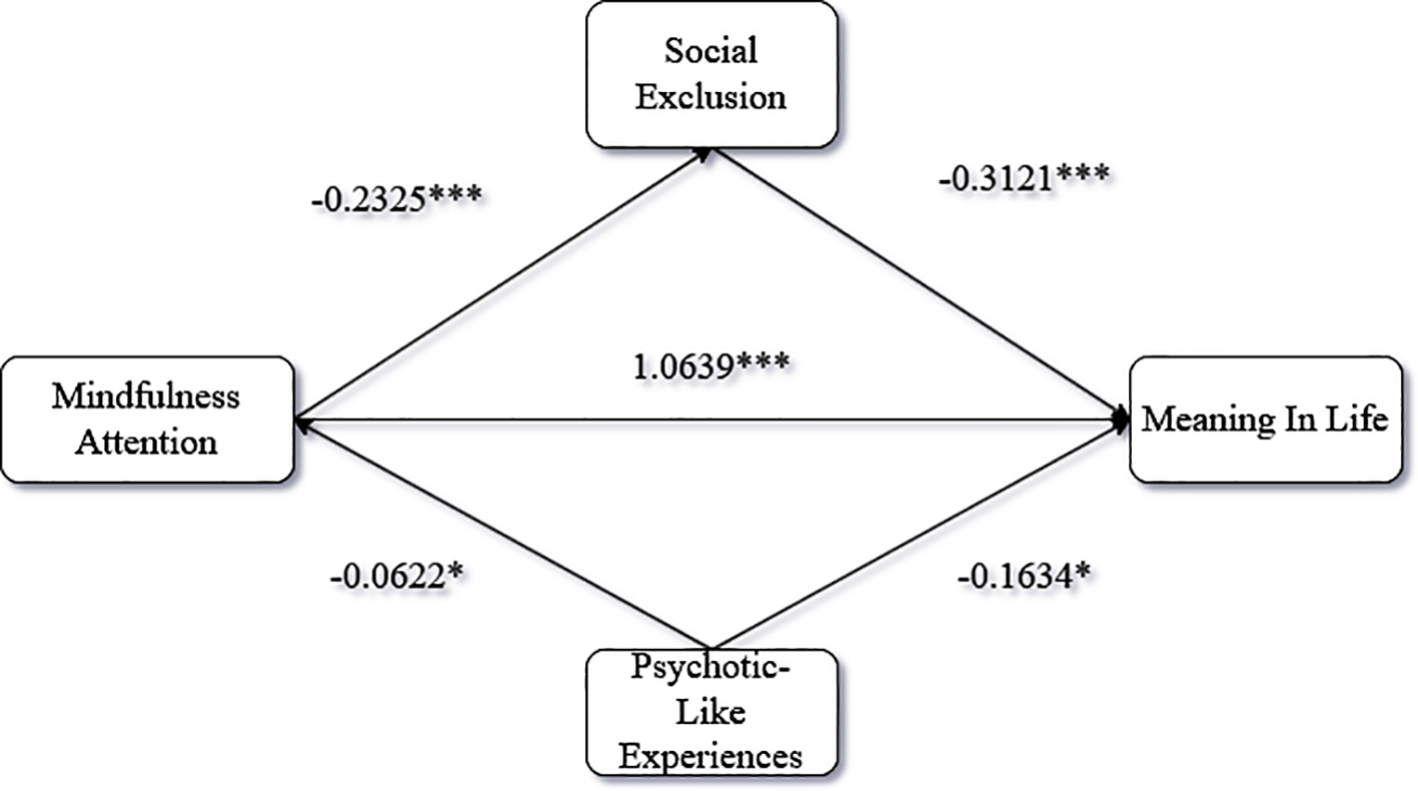
PROCESS model results.

To test the mediating roles of social exclusion and psychotic-like experiences in the link between mindfulness attention and meaning in life, we utilized Hayes’ PROCESS macro (Model 4). The analysis yielded significant results consistent with our hypotheses. Mindfulness attention was negatively associated with both proposed mediators: social exclusion (β = -0.2325, 95% CI [-0.3513, -0.1138]) and social anhedonia (β = -0.0622, 95% CI [-0.1225, -0.0019]). Subsequently, both social exclusion (β = -0.3121, 95% CI [-0.3838, -0.2404]) and social anhedonia (β = -0.1634, 95% CI [-0.3045, -0.0222]) negatively predicted meaning in life, even after accounting for the strong, positive direct effect of mindfulness attention (β = 1.0639, 95% CI [0.9417, 1.1861]). Most importantly, the specific indirect pathways were confirmed as significant. The mediating effect of social exclusion was robust (Effect = 0.0726, 95% CI [0.0384, 0.1119]), and the mediation via social anhedonia was also significant (Effect = 0.0102, 95% CI [0.0000, 0.0260]), as its confidence interval did not cross zero **Figure 3**.

## 5 General discussion

### 5.1 Research summary

The current study provided robust empirical validation for all five of its hypotheses. We first confirmed a significant positive association between digital mindfulness training and social workers’ meaning in life (H1). More importantly, a parallel mediation model was supported (H2 & H3), revealing how this effect operates: training first reduced perceived social exclusion, which in turn lessened psychotic-like experiences (46). Complementing this mechanistic insight, our analysis confirmed the existence of five distinct and meaningful latent profiles (H4): the Flourishing Professional, the Socially Isolated yet Resilient, the Typical Practitioner, the Internally Struggling but Connected, and the High-Risk and Distressed. Finally, as predicted (H5), these profiles exhibited significantly different levels of meaning in life, with the High-Risk/Distressed profile reporting the lowest (47). These integrated, validated findings offer a comprehensive framework for developing targeted interventions.

### 5.2 Theoretical contributions

This study makes several theoretical contributions. First, our study significantly extends the application of Conservation of Resources (COR) theory by translating its abstract principles into a concrete, empirically-derived typology of professional well-being (48). While COR theory provides a powerful lens for understanding resource dynamics, its core concepts of ‘resource caravans’ and ‘gain/loss spirals’ have predominantly been examined through variable-centered approaches. Such methods capture the average trend but often obscure the crucial heterogeneity within a population (49). Our use of Latent Profile Analysis directly confronts this limitation. By identifying five distinct psychosocial profiles among social workers, we provide a tangible manifestation of how resource ecologies are configured in a real-world professional population, illustrating the holistic patterns of resource clustering that the theory describes (50). This research marks a significant step forward for both organizational psychology and the study of professional well-being. By introducing a clear typological framework, we challenge the limitations of simplistic, linear models of stress and coping. This approach provides a practical diagnostic map, allowing for the identification of distinct practitioner subgroups (51), each with its own profile of resources and vulnerabilities. In essence, we offer a more refined method for understanding how the dynamics of personal and professional resources play out within complex organizational settings (52).

Second, our findings challenge the long−held view of social workers as a homogeneous body. Much prior research isolates variables such as burnout or supervision, overlooking the heterogeneity in lived adaptation (53). Our five profiles make this diversity visible, particularly the “Socially Isolated yet Resilient” (C2) and the “Internally Struggling but Connected” (C4) groups. The C2 profile reveals a group that maintains robust mental health (low PLEs) despite experiencing significant professional exclusion (high SE). This form of adaptation complicates linear stress-strain models, suggesting that internal psychological resources can serve as a powerful buffer against a negative social environment (54). Conversely, the C4 profile presents a different paradox: social integration (low SE) co-existing with significant internal distress (high PLEs). This reveals that resilience is not merely the absence of social stressors; one can appear well-connected yet be privately battling significant mental health challenges (55). By framing these patterns within an evidence-based typology, we expand theoretical accounts of professional adaptation, accommodating pathways that are resilient despite marginalization, or internally distressed despite social connection.

Finally, this study delineates two distinct micro-pathways by which external social stressors and internal psychological states can independently erode a meaning in life, offering a more nuanced account of resource management within COR theory (56). Our parallel mediation model reveals that a single intervention can operate on two separate fronts. The first pathway demonstrates how mindfulness training acts as a protective buffer against the erosion of social-relational resources. By mitigating the perception of social exclusion (SE), the intervention prevents a primary resource loss that directly threatens an individual’s sense of belonging and professional esteem (57). Protecting this foundational social resource is, in itself, a critical route to preserving meaning. Concurrently, the second, independent pathway reveals a mechanism for preserving internal cognitive resources. Mindfulness training was also shown to reduce psychotic-like experiences (PLEs), thereby quieting the cognitive static that can destabilize one’s sense of reality and undermine the mental scaffolding needed to sustain a coherent life narrative. This finding refines COR theory by illustrating that the erosion of meaning is not a single cascade but can result from distinct social and cognitive vulnerabilities. The contribution extends to clinical occupational psychology and stress research by demonstrating that safeguarding meaning in high-stress roles requires a dual focus: it is as much a cognitive imperative to maintain internal stability as it is a social imperative to foster belonging (58).

### 5.3 Practical implications

This study yields several practical and managerial implications. First, the findings challenge the efficacy of traditional one-size-fits-all well-being programs and suggest a necessary shift toward precision-based, tiered support systems for social workers. The identification of five distinct profiles provides a strong diagnostic framework to drive this transition. For instance, rather than providing a generic wellness app to all employees, organizations could implement short, confidential annual screenings using validated scales for social exclusion (SE) and psychotic-like experiences (PLEs). This approach would allow for the identification of each employee’s profile, enabling a tiered, need-aligned support plan. For employees in the ‘Socially Isolated yet Resilient’ group, organizations could offer digital resources focused on navigating difficult interpersonal dynamics and leveraging their internal resilience. In contrast, identifying an employee in the ‘High-Risk/Distressed’ profile should trigger immediate and confidential outreach from a trained wellness officer, including direct access to counseling and a caseload review. Such a strategy transforms support from a passive, optional perk into an active, data-driven system that directs the most intensive help where it is most critically needed.

Second, our mechanistic findings provide a clear blueprint for designing dual-function digital mindfulness interventions that are both protective and generative. The parallel mediation pathways show that simply teaching relaxation is insufficient; the training must target the specific mechanisms of resource loss and gain (59). An effective intervention should therefore be structured into two complementary streams. A “Defensive Shield” stream would focus on mitigating the impact of social exclusion, incorporating specific exercises like the RAIN meditation for processing difficult interpersonal events and “thought defusion” techniques to de-personalize criticism. Concurrently, a “Generative Catalyst” stream would actively build positive resources through practices such as a “Three Good Things in My Work” guided journal to savor client successes and Values-Based Intention Setting meditations to connect daily tasks to a core sense of professional meaning (22). This dual-focus design ensures that the intervention not only helps social workers weather storms but also equips them to find the sun.

Finally, this research argues for a systemic evolution from reactive care to proactive organizational and educational immunization. Addressing distress cannot be the sole responsibility of the individual; the system must share the burden (60). For organizations, this means implementing policies that directly reduce the sources of social exclusion. This should include mandatory manager training focused on providing psychologically safe feedback and facilitating inclusive team dynamics, as well as revising supervision models to formally dedicate time to discussing the practitioner’s emotional well-being, not just administrative tasks (61). This dedicated time becomes the crucial juncture for deploying tailored support. For instance, a supervisor could guide a practitioner identified as ‘Striving-but-Struggling’ toward digital modules focused on preventative tools like value clarification exercises and stress management techniques to reinforce their purpose before it erodes. Conversely, for a colleague in the ‘High-Risk/Distressed’ group, the same supervisory check-in would trigger a different pathway, recommending a digital intervention that prioritizes stabilizing content—such as guided self-compassion and grounding exercises—while crucially embedding immediate links to formal clinical care. For social work education, this translates to embedding “psychological personal protective equipment” (PPE) as a core competency. Curricula should integrate mandatory, semester-long experiential courses on emotional resilience and mindfulness. A powerful capstone requirement could be the development of a “Professional Sustainability Plan,” (62) where students articulate their personal strategies for managing stress and seeking support, thus equipping the next generation with the tools for a long and meaningful career from day one.

### 5.4 Limitations and future research

This study has several limitations that warrant consideration. First and foremost, our reliance on a convenience sampling method, recruiting from online professional communities and social media groups, introduces a significant potential for selection bias. This approach may have resulted in a sample that is not fully representative of the broader social worker population in China. For instance, participants recruited online are likely to be more technologically adept, potentially younger, and more actively engaged in seeking peer support via digital platforms. Conversely, this method could have systematically excluded social workers who are less active online, such as those in older age brackets or those working in rural areas. Consequently, the generalizability of our findings, particularly the prevalence and characteristics of the five latent profiles, should be interpreted with caution. Future research should aim to replicate these findings using more robust sampling strategies, such as stratified random sampling from official professional registries, to ensure the sample more accurately reflects the heterogeneity of the entire profession. Second, the study’s cross-sectional design reveals correlations but cannot establish causality; future longitudinal research is needed to explore the dynamic relationships over time (63). Third, while the sample was exclusively Chinese, which limits generalizability to other cultural contexts, the aforementioned sampling issue poses a more immediate challenge to its generalizability even within China. Finally, the reliance on self-report data may be subject to social desirability bias; future studies could benefit from incorporating behavioral observations or multi-source data for triangulation. In addition, participation in digital mindfulness programs may be subject to self-selection bias, whereby individuals with greater prior interest in mindfulness, higher intrinsic motivation, or higher digital literacy are more likely to enroll. This could further limit the generalizability of our findings to practitioners who are less engaged with or less inclined toward digital mindfulness interventions.

## Data Availability

The raw data supporting the conclusions of this article will be made available by the authors, without undue reservation.

## References

[1] Lu BY Onoye JM Ba KMK Toohey TP Takeshita J . Impact of psychiatric social workers on length of stay in psychiatric emergency service. Acad Emergency Med. (2024) 31:3. doi: 10.1111/acem.14808, PMID: 37739801

[2] Jay EK Moxham L Roberts M Yousiph T Robson G Lewer K . Contributing to ‘a sense of purpose’ – Evaluating consumer recovery progress after attending a therapeutic-recreation intervention program: A quantitative analysis. Int J Soc Psychiatry. (2024) 70:926–32. doi: 10.1177/00207640241242024, PMID: 38605480 PMC11323423

[3] Liu C Chen H Liu CY Lin R Chiou WK . Effects of Loving-Kindness Meditation on Mindfulness, Spirituality and Subjective Weil-Being of Flight Attendants. Cham: Springer (2020). doi: 10.1007/978-3-030-49913-6_13 PMC7349275 32560125

[4] Sercekman MY Akca M . Transforming managers with mindfulness-based training: a journey towards humanistic management principles. Curr Psychol. (2025). doi: 10.1007/s12144-024-07260-2

[5] Parlikar N Strand LB Kvaly K Espnes GA Moksnes UK . The prospective association of adolescent loneliness and low resilience with anxiety and depression in young adulthood: The HUNT study. Soc Psychiatry Psychiatr Epidemiol. (2025) 60:2223–35. doi: 10.1007/s00127-025-02888-2, PMID: 40195157 PMC12378919

[6] Li J Li L Zhao S . Predator-prey survival pressure is sufficient to evolve swarming behaviors. New J Phys. (2023) 25. doi: 10.1088/1367-2630/acf33a

[7] Liu A Zhou G . The intervention effect of online mindfulness training in alleviating youths’ Test anxiety. Youth Soc. (2025) 57:117–38. doi: 10.1177/0044118X241248476

[8] Palmer D Fan N . Achievements, free will, and meaning in life. Synthese. (2024) 204. doi: 10.1007/s11229-024-04791-w

[9] Liebherr M Brandtner A Brand M Tang YY . Digital mindfulness training and cognitive functions: A preregistered systematic review of neuropsychological findings. Ann New York Acad Sci. (2024) 1532. doi: 10.1111/nyas.15095, PMID: 38197226

[10] Zhao Z Gao H Chen F Dong H . The mediating effect of job burnout on perceived stress and presenteeism among geriatric caregivers in long-term care facilities. Geriatric Nurs. (2025) 61:538–43. doi: 10.1016/j.gerinurse.2024.12.007, PMID: 39742542

[11] Hu Q Lu YB Pan Z Wang B . How does AI use drive individual digital resilience? a conservation of resources (COR) theory perspective. Behav Inf Technol. (2022). doi: 10.1080/0144929x.2022.2137698

[12] Chen WR Whalen DH Tiede MK . A dual mechanism for intrinsic f0. J Phonetics. (2021) 87:101063–. doi: 10.1016/j.wocn.2021.101063, PMID: 34012182 PMC8128133

[13] Wang X Iqbal S Amin N Hussain M Zaman S Khan S . The role of government effectiveness, technological innovations, natural resource protection on carbon emissions in Gulf Cooperation Council region: A pathway for achieving sustainable development goals by 2030. J Environ Manage. (2025) 377. doi: 10.1016/j.jenvman.2025.124506, PMID: 39999751

[14] Yael A Eran L Orly N Naama W Eran Z . COR and CORONA: analysis of COVID-19’s subjective lasting impact on wellbeing, employing conservation of resources theory. J Public Health. (2025). doi: 10.1093/pubmed/fdaf021, PMID: 39957434

[15] Tanabe M Kunisawa K Saito I Kosuge A Tezuka H Kawai T . The spatial cognitive dysfunction and microglial activation induced by adolescent social isolation through the hypoproduction of cystine by the damage of colonic goblet cells. Int J Neuropsychopharmacol. (2025) 28. doi: 10.1093/ijnp/pyae059.617

[16] Im EO . Research funding, social responsibilities, and nursing knowledge generation. Adv Nurs Sci. (2025) 48:115–6. doi: 10.1097/ANS.0000000000000563, PMID: 40198833

[17] Cao Z Mustafa M Isa MHM . Traditional regionalism or modern minimalism? Unveiling the psychological impact of architectural styles in sustainable urban planning. Sustainability. (2024) 16:5576. doi: 10.3390/su16135576

[18] Jiang Y Cao Y . Effects of physical exercise on college students’ meaning in life in life: the chain mediating role of stress perception and mental toughness. Front Psychol. (2025). doi: 10.3389/fpsyg.2025.1612957, PMID: 40535180 PMC12174108

[19] Mitchell JT Davis NO Lunsford-Avery JR . Trait mindfulness in adolescents with and without Attention-Deficit/Hyperactivity Disorder. Res Dev Disabil. (2025) 158. doi: 10.1016/j.ridd.2025.104926, PMID: 39892034 PMC11969500

[20] Barclay K Fabinyi M Song AY Ota Y Vandenberg J Mcclean N . What are the impacts on community wellbeing of social relations in conservation projects? Conserv Soc. (2024) 22. doi: 10.4103/cs.cs_103_22

[21] Compare C Lorusso MM Albanesi C . The power of connection: Resource and responsibility in the virtual community experience of Italian trans and gender-diverse activists. J Community Appl Soc Psychol. (2024) 34. doi: 10.1002/casp.2859

[22] Liu C Chen H Liang YC Lin R Chiou WK . ISDT Case Study of Loving Kindness Meditation for Flight Attendants. Cham: Springer (2021). doi: 10.1007/978-3-030-77077-8_16

[23] Zhang P Wang Y You Y Yuan J Zhou Z Sun S . Generation pathway of hydroxyl radical in Fe/N/C-based oxygen reduction electrocatalysts under acidic media. J Phys Chem Lett. (2021) 12:7797–803. doi: 10.1021/acs.jpclett.1c01905, PMID: 34375530

[24] Liu C Chen H Zhang A Gong X Wu K Liu C-Y . The effects of short video app-guided loving-kindness meditation on college students’ mindfulness, self-compassion, positive psychological capital, and suicide ideation. Psicologia: Reflexão e Crítica. (2023) 36:32. doi: 10.1186/s41155-023-00276-w, PMID: 37902928 PMC10616025

[25] Lu Y Lin S Shen ZJM Zhang J . Location planning, resource reallocation and patient assignment during a pandemic considering the needs of ordinary patients. Health Care Manage Sci. (2025) 28:234–58. doi: 10.1007/s10729-025-09703-z, PMID: 40347358

[26] Figueroa E Lucero JE Rodríguez JE . Watch your career like you watch your money: minority tax mitigation strategies. South Med J. (2025) 118:346–8. doi: 10.14423/SMJ.0000000000001834, PMID: 40456549

[27] Salajan FD Jules TD . The global resurgence of authoritarianism and its existential threats to education: implications for scholarship in comparative and international education. Comp Educ Rev. (2024) 68:26. doi: 10.1086/732119

[28] Chen H Liu C Hsu S-E Huang D-H Liu C-Y Chiou W-K . The effects of animation on the guessability of universal healthcare symbols for middle-aged and older adults. Hum Factors: J Hum Factors Ergonomics Soc. (2023) 65:1740–58. doi: 10.1177/00187208211060900, PMID: 34969321

[29] Yilmaz O Harma M Doruyol B . Validation of morality as cooperation questionnaire in Turkey, and its relation to prosociality, ideology, and resource scarcity. Eur J psychol Assess. (2021) 37:149–60. doi: 10.1027/1015-5759/a000627

[30] Zhang H Jing Z Ali S Asghar M Kong Y . Renewable energy and natural resource protection: Unveiling the nexus in developing economies. J Environ Manage. (2024) 349. doi: 10.1016/j.jenvman.2023.119546, PMID: 37976646

[31] Gross M . Recovering a sense of reality. Curr Biol. (2021) 31:R1–3. doi: 10.1016/j.cub.2020.12.008

[32] Daneshvar S Shafiei M Basharpoor S . Group-based compassion-focused therapy on experiential avoidance, meaning-in-life, and sense of coherence in female survivors of intimate partner violence with PTSD: A randomized controlled trial. J Interpersonal Violence. (2022) 37:NP4187–211. doi: 10.1177/0886260520958660, PMID: 32933348

[33] Hamilton CA Matthews FE Donaghy PC Taylor JP O’Brien JT Barnett N . Prospective predictors of decline v. stability in mild cognitive impairment with Lewy bodies or Alzheimer’s disease. psychol Med. (2021). doi: 10.1017/s0033291720001130, PMID: 32366348

[34] Chiou W-K Liu C Chen H Hsu S-E . Reliability and validity assessment of the Chinese version of flow ergonomics. In: Rau P-LP , editor. Cross-Cultural Design. Interaction Design Across Cultures. Lecture Notes in Computer Science Springer International Publishing, Cham (2022). p. 330–41. p. 13311.

[35] Monov G Stein H Klock L Gallinat J Kühn S Lincoln T . Linking cognitive integrity to working memory dynamics in the aging human brain. J Neurosci. (2024) 44:20. doi: 10.1523/JNEUROSCI.1883-23.2024, PMID: 38760163 PMC11211717

[36] Li Y . Inferring meaningful change in quality of life with posterior predictive distribution: an alternative to standard error of measurement. Qual Life Res. (2022), 1–10. doi: 10.1007/s11136-022-03239-3, PMID: 36083421 PMC10202216

[37] Zhaoyang X Jingyue Z Zhifa L . The relationship between work and family interference and service quality among Chinese social workers: the roles of emotional exhaustion and work support. Br J Soc Work. (2024). doi: 10.1093/bjsw/bcae088

[38] Gunen B Stott D Milliron BJ Butryn M Harhay MN Klassen AC . Patient perspectives on social support-related facilitators and barriers to kidney diet adherence: A photovoice study. J Am Soc Nephrol. (2024) 35. doi: 10.1681/ASN.2024w6ajb3kh

[39] Yang F Huang X Tang D Yang J Wu L . the international journal of human resource management how guanxi hrm practice relates to emotional exhaustion and job performance: the moderating role of individual pay for performance. Int J Hum Resource Manage. (2021) 32:2493–518. doi: 10.1080/09585192.2019.1588347

[40] Ahmed S Islam MS Antu UB Islam MM Rajput VD Mahiddin NA . Nanocellulose: A novel pathway to sustainable agriculture, environmental protection, and circular bioeconomy. Int J Biol Macromolecules. (2025) 285. doi: 10.1016/j.ijbiomac.2024.137979, PMID: 39592042

[41] Davidson RJ Kabat-Zinn J Schumacher JMS Rosenkranz MBA Muller DMD Santorelli SF . Alterations in Brain and Immune Function Produced by Mindfulness Meditation. Psychosomatic Medicine. (2003) 65(4):564–70. doi: 10.1097/01.PSY.0000077505.67574.E3, PMID: 12883106

[42] Brown RP . Measuring individual differences in the tendency to forgive: construct validity and links with depression. Pers Soc Psychol Bull. (2003) 29:759–71. doi: 10.1177/0146167203029006008, PMID: 15189631

[43] Hinterbuchinger B Mossaheb N . Psychotic-like experiences: a challenge in definition and assessment. Front Psychiatry. (2021) 12:582392. doi: 10.3389/fpsyt.2021.582392, PMID: 33854445 PMC8039445

[44] Gilman R Carter-Sowell A DeWall CN Adams RE Carboni I . Validation of the ostracism experience scale for adolescents. psychol Assess. (2013) 25:319. doi: 10.1037/a0030913, PMID: 23205625

[45] Steger MF Frazier P Oishi S Kaler M . The meaning in life questionnaire: assessing the presence of and search for meaning in life. J Couns Psychol. (2006) 53:80. doi: 10.1037/0022-0167.53.1.80

[46] Ren SY Sun ZL Yang J . The use of biochemical indexes in hair for clinical studies of psychiatric diseases: What can we learn about mental disease from hair? J Psychiatr Res. (2023) 158:305–13. doi: 10.1016/j.jpsychires.2023.01.004, PMID: 36628872

[47] Mahmoodabadi M Khoshnood Z Khandani BK . The relationship between body image and meaning of life among women with breast cancer in Kerman, Iran. Cancer Invest. (2024) 42:9. doi: 10.1080/07357907.2024.2371369, PMID: 39109710

[48] Shelef L Schiff M Pat-Horenczyk R Dekel R . COVID-19 vs. terrorism: Contribution of the COR theory to the process of coping with invisible threats. J Psychiatr Res. (2022) 147:176–82. doi: 10.1016/j.jpsychires.2022.01.023, PMID: 35051716 PMC8753990

[49] Chaudhury SN Ding E Jespersen NE Onuchic JN Sanbonmatsu KY . Conformational heterogeneity in the dGsw purine riboswitch: role of Mg and 2’-dG in aptamer folding. RNA. (2025) 31. doi: 10.1261/rna.080274.124, PMID: 40021341 PMC12001964

[50] Schoenecker KA Esmaeili S King SRB . Seasonal resource selection and movement ecology of free-ranging horses in the western United States. J Wildlife Manage. (2023) 87. doi: 10.1002/jwmg.22341

[51] Garin T Arfib B Ladouche B Goncalves J Dewandel B . Improving hydrogeological understanding through well-test interpretation by diagnostic plot and modelling: a case study in an alluvial aquifer in France. Hydrogeology J. (2022) 30:20. doi: 10.1007/s10040-021-02426-9

[52] Tan F Lyu D . Exploring healthy green practices that influence customers’ Green engagement value in restaurants. Soc Behav Personality: Int J. (2025) 53:1–8. doi: 10.2224/sbp.13261

[53] Hojjat NM Wahyu SWA Andrew Y Garrett C Cornelius VA Clare-Ann N . Unveiling impaired vascular function and cellular heterogeneity in diabetic donor-derived vascular organoids. Stem Cells. (2024) 9. doi: 10.1093/stmcls/sxae043, PMID: 39049437 PMC11384901

[54] Higdon AL Brar GA . Rules are made to be broken: a “simple” model organism reveals the complexity of gene regulation. Curr Genet. (2021) 67:1–8. doi: 10.1007/s00294-020-01121-8, PMID: 33130938 PMC7887019

[55] Dawson ST . Refugee children and the emotional cost of internationalism in interwar Britain. J Br Stud. (2021) 60:115–39. doi: 10.1017/jbr.2020.189

[56] Ali Z Mehreen A . Can you manage shocks? An investigation of career shocks on proactive career behavior: a COR theory perspective. J Managerial Psychol. (2022) 37. doi: 10.1108/JMP-04-2020-0206

[57] Xu W Long J Liu J Luo H Duan H Zhang Y . A novel porous polyimide membrane with ultrahigh chemical stability for application in vanadium redox flow battery. Chem Eng J. (2022) 428:131203–. doi: 10.1016/j.cej.2021.131203

[58] Garmaise-Yee JS Montague J Mallei G Hall BLM Williams AS . Sense of belonging for BIPOC nursing students: A participatory action research study. J Nurs Educ. (2025) 64. doi: 10.3928/01484834-20250129-06, PMID: 40332996

[59] Tschenett H Vafai-Tabrizi F Zwick RH Valipour A Funk GC Nater UM . Late Breaking Abstract - A digital mindfulness-based intervention improves mental health in COPD - Findings from a pilot RCT. Eur Respir J. (2024) 64:2. doi: 10.1183/13993003.congress-2024.OA1880

[60] Perlmutter JI Chapman JR Wilkinson MC Nevarez-Saenz I Unckless RL . A single amino acid polymorphism in natural Metchnikowin alleles of Drosophila results in systemic immunity and life history tradeoffs. PloS Genet. (2024) 20. doi: 10.1371/journal.pgen.1011155, PMID: 38466751 PMC10957085

[61] Xiao Q Cao Y Wu S Zou Y Hu X . Can positive emotional writing improve the emotional health level of international medical students? Evidence from a randomized controlled trial. BMC Med Educ. (2024) 24:1–9. doi: 10.1186/s12909-024-06186-4, PMID: 39487490 PMC11529312

[62] Chang LA . Designing and implementing sustainable professional development programs: embodied curriculum and instruction for kindergarten teachers. Sustainability. (2024) 16. doi: 10.3390/su16177327

[63] Condominas E Sanchez-Niubo A Domènech-Abella J Haro JM Bailon R Giné-Vázquez I . Exploring the dynamic relationships between nocturnal heart rate, sleep disruptions, anxiety levels, and depression severity over time in recurrent major depressive disorder. J Affect Disord. (2025) 376. doi: 10.1016/j.jad.2025.02.010, PMID: 39922289

